# Local variance of atmospheric ^14^C concentrations around Fukushima Dai-ichi Nuclear Power Plant from 2010 to 2012

**DOI:** 10.1007/s10967-017-5459-8

**Published:** 2017-09-08

**Authors:** Biying Chen, Sheng Xu, Gordon T. Cook, Stewart P. H. T. Freeman, Xiaolin Hou, Cong-Qiang Liu, Philip Naysmith, Katsuhiko Yamaguchi

**Affiliations:** 1 Scottish Universities Environmental Research Centre (SUERC), East Kilbride, G75 0QF UK; 2 Institute of Surface-Earth System Science, Tianjin University, Tianjin, 300072 China; 3 Faculty of Symbiotic Systems Science, Fukushima University, Fukushima, 960-1296 Japan; 4 Center for Nuclear Technologies, Technical University of Denmark, 4000 Roskilde, Denmark

**Keywords:** Tree rings, Radiocarbon, Fukushima Nuclear Reactor accident

## Abstract

Radiocarbon (^14^C) has been measured in single tree ring samples collected from the southwest of the Fukushima Dai-ichi Nuclear Power Plant. Our data indicate south-westwards dispersion of radiocarbon and the highest ^14^C activity observed so far in the local environment during the 2011 accident. The abnormally high ^14^C activity in the late wood of 2011 ring may imply an unknown source of radiocarbon nearby after the accident. The influence of ^14^C shrank from 30 km during normal reactor operation to 14 km for the accident in the northwest of FDNPP, but remains unclear in the southwest.

## Introduction

The 2011 Fukushima Dai-ichi Nuclear Power Plant (FDNPP) accident resulted in the release of a huge amount of radioactive materials to the atmosphere. Estimated total release reached 5.2 × 10^17^ Bq [1]. Following the accident, much work has been done to investigate issues such as levels, chemical behaviour and long-term variations in the released radionuclides within the local and worldwide environments. Due to the concentrated research on major volatile or short-lived radionuclides, such as ^131^I, ^129^Te, ^132^Te, ^134^Cs, ^137^Cs, etc. [2–8], the limited information of other released long-lived radionuclides, like ^14^C, constrains the understanding of their influence on the environment and making a comprehensive evaluation of the Fukushima accident [1].

^14^C is naturally produced by the nuclear reaction ^14^N(n,p)^14^C (and less so by ^13^C(n,γ)^14^C and ^17^O(n,α)^14^C) [1]. Atmospheric ^14^C activity has been enhanced by the nuclear weapon tests in the 1950s and 1960s, discharges associated with the nuclear fuel cycle and nuclear accidents, and diluted by ^14^C-free fossil fuel combustion from biosphere [9, 10]. To study how these factors influence the atmospheric ^14^C activity, tree rings are considered an ideal sample type because of their rapid response to atmospheric ^14^C variability [11–13].

A previous study of ^14^C released from the Fukushima accident has revealed that the ^14^C excess was likely distributed along a northwest direction from FDNPP, and declined quickly with increasing distance [14]. This pattern is consistent with that of the short half-life radionuclides (^131^I, ^134^Cs, etc.) that also mainly dispersed north-westwards [6, 15]. No ^14^C excess was found in 2011 tree ring samples from Okuma (OKU, 1 km southwest of FDNPP) [16] and Iwaki (IWA, 50 km south of FDNPP) [10], which made it unclear whether any released ^14^C had been dispersed southwards. The major dispersion of the radionuclides during the accident was on the 15th and 21st March, mainly controlled by the wind direction and precipitation [5]. The dominant wind direction was from the southwest (15th–16th March) and northeast (21st–23rd March) [5]. Adachi et al. [7] detected two significant peaks of Cs-bearing particles in Tsukuba, 170 km southwest of FDNPP while similar observations that radioactive materials were also present to the southwest of FDNPP have been reported [2, 5]. All the information here supports the fact that radionuclides were dispersed to the south of FDNPP, which is distinct from the previous study on ^14^C [16].

**Fig. 1:**
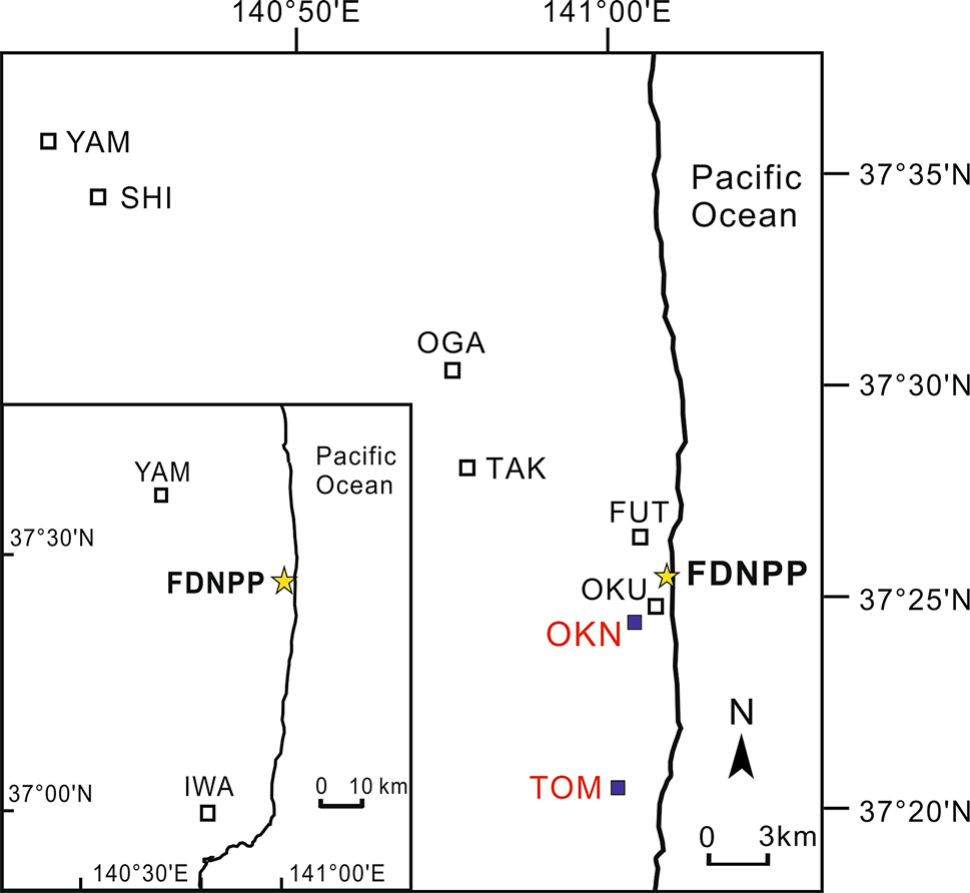
Map showing sampling site in the vicinity of the FDNPP. The *solid* and *open squares* represent the new sampling sites and the previous sampling locations [14, 16], respectively. *IWA* Iwaki, *YAM* Yamakiya, *SHI* Shimotsushima, *OKA* Ogaki, *TAK* Takase, *FUT* Futuba, *OKU* Okuma, *OKN* Okuma-N, *TOM* Tomioka

In the present study, to identify and confirm whether there was any ^14^C excess to the southwest of FDNPP, and re-evaluate the ^14^C emission caused by the accident, we analyse new tree ring samples from cedar trees at Okuma-N (OKN, 37°24′27.6″N, 141°00′56.8″E), 2 km southwest of FDNPP and Tomioka (TOM, 37°20′33.0″N, 141°00′16.5″E), 9 km southwest of FDNPP (Fig. 1). In addition, considering the possibility that the excess ^14^C in 2011 tree ring from Okuma (OKU), published in [16], might be masked by the annual mixing, a duplicate tree ring sample was analysed to distinguish the intra-annual variance of ^14^C in 2011, which would also enable us to examine the reproducibility of the results.

## Experimental

The samples used in this study are tree cores from Japanese cedar (*Cryptomeria japonica*), collected on 19th May 2016 by a tree ring corer with a 1 cm diameter. For each location, two trees were cored. The diameters of the tree trunks at 1 m height were 40, 105, 97 and 106 cm for OKN-1, OKN-2, TOM-1 and TOM-2, respectively. The duplicate sample from OKU is the same tree species. The details of the duplicate sample from OKU was described elsewhere [16]. The tree rings from 2010 to 2012 were identified and sectioned off separately (the tree ring for the 2010 in the OKN-1 sample was not measured). Meanwhile, the early and late wood units of tree rings from 2010 to 2011 were divided to explore the sub-annual change.

The alpha-cellulose fraction was extracted for ^14^C measurement [17]. The detailed procedures and conditions for alpha-cellulose extraction, combustion and graphitization are described in [18]. A brief description is as follows. The individual samples were refluxed with chloroform/ethanol (2:1 by volume), ethanol, and water, each for 8 h. This was followed by a bleaching step with hypochlorite solution and finally, acid–base-acid steps to isolate a pure alpha-cellulose fraction [16]. The extracted cellulose was combusted in sealed, evacuated quartz tubes containing copper oxide and silver foil at 850 °C. An aliquot of CO_2_ was used for stable isotope analysis for each sample. The δ^13^C was determined by conventional isotope ratio mass spectrometer (IRMS) using a VG SIRA 11. The results are expressed as per mil deviations from the Vienna Pee Dee Belemnite (VPDB) standard and have an uncertainty of ±0.1‰. Another aliquot of CO_2_ was catalytically reduced to pure graphite by reacting with Zn and Fe. The graphite was then pressed into 1 mm internal-diameter aluminium sample holders and loaded into a 5 MV accelerator mass spectrometer (AMS) for ^14^C/^13^C measurements [19]. To assess the uncertainty and ^14^C background during this analysis, in-house humic acid secondary standard samples (VIRI Sample T) [20] and interglacial wood samples (Heidelberg wood) were measured along with the tree ring samples. The uncertainty of all ^14^C measurement results reached better than 2‰ for both the ^14^C counting statistics and the repeat measurement scatter. The IRMS measured δ^13^C was used to correct for isotopic fractionation. The ∆^14^C is expressed as per mil derivations from the primary standard sample (0.7459 times activity of NBS oxalic acid II (SRM-4990C)).

## Results and discussion

### Excess ^14^C southwest of FDNPP in 2011

The results of radiocarbon and stable carbon isotope measurements on the samples are listed in Table 1 together with the published data for the 2010–2012 tree-ring samples from OKU [16]. To estimate the change in ∆^14^C within the vicinity of Fukushima, caused by the nuclear accident, the atmospheric ∆^14^C data from monitoring station in Schauinsland (SCL), Black Forest, Germany (47°55′N, 7°54′E) [21] was chosen as the background value as in previous studies [10, 14, 16] for the lack of direct information of that in Japan. However, the disparity in latitude between FDNPP and SCL might cause a different background value for variant emissions of ^14^C-free CO_2_ [9]. Besides, the FDNPP is on the western coast of the Pacific Ocean, different from SCL (inland), which might affect the ^14^C concentration in the atmosphere through ocean–atmosphere exchange. Considering these factors, we examined available ∆^14^C data of air samples over the period 1992–2007 at La Jolla (LAJ), California (32.87°N, 117.25°W) [9] which is on the eastern coast of the Pacific Ocean and a relatively similar environmental situation to FDNPP. The time from May to August was suggested to represent the biogenic conversion period [10, 21]. The ∆^14^C data during this time from 2000 to 2007 was taken and averaged to compare with the values from SCL. The difference between these two sets of values (LAJ and SCL) was less than 4‰. Such a small difference supported the choice of using the measured data in 2010–2012 from SCL as the northern-hemispheric ^14^C background value.

**Table 1:** Analytical ^14^C activity of tree rings from Fukushima, Japan

Lab code (SUERC-)	Year	Wood fraction	δ^13^C (‰)	F^14^C (fraction modern)	∆^14^C (‰)	Background ∆^14^C (‰)	Excess ∆^14^C (‰)
1. Okuma (OKU, 37°24′47.1″N, 141°01′34.7″E, SW 1 km of FDNPP)							
63065^a^	2012	Whole ring	−26.1	1.0476 ± 0.0022	47.6 ± 2.2	31.5 ± 2.0	16.1 ± 4.2
63066^a^	2011	Whole ring	−26.3	1.0403 ± 0.0020	40.3 ± 2.0	39.0 ± 2.4	1.3 ± 4.4
68802	2011	Late ring	−25.8	1.0354 ± 0.0019	35.4 ± 1.9	39.0 ± 2.4	−3.6 ± 4.3
68803	2011	Early ring	−25.0	1.0395 ± 0.0022	39.5 ± 2.2	39.0 ± 2.4	0.5 ± 4.6
63067^a^	2010	Whole ring	−25.8	1.1803 ± 0.0023	180.3 ± 2.3	40.0 ± 2.7	140.3 ± 5.0
68804	2010	Late ring	−25.5	1.1804 ± 0.0025	180.4 ± 2.5	40.0 ± 2.7	140.4 ± 5.2
68805	2010	Early ring	−26.2	1.1230 ± 0.0021	123.0 ± 2.1	40.0 ± 2.7	83.0 ± 4.8
2. Okuma-N-1 (OKN-1, 37°24′27.6″N, 141°00′56.8″E, SW 2 km of FDNPP)							
70660	2012	Whole ring	−22.7	1.0435 ± 0.0021	43.5 ± 2.1	31.5 ± 2.0	12.0 ± 4.1
70661	2011	Late ring	−23.0	1.2864 ± 0.0026	286.4 ± 2.6	39.0 ± 2.4	247.4 ± 5.0
70666	2011	Early ring	−22.6	1.2266 ± 0.0025	226.6 ± 2.5	39.0 ± 2.4	187.6 ± 4.9
3. Okuma-N-2 (OKN-2, 37°24′27.6″N, 141°00′56.8″E, SW 2 km of FDNPP)							
68772	2012	Whole ring	−24.7	1.0454 ± 0.0022	45.4 ± 2.2	31.5 ± 2.0	13.9 ± 4.2
68774	2011	Late ring	−24.3	1.3121 ± 0.0028	312.1 ± 2.8	39.0 ± 2.4	273.1 ± 5.2
68773	2011	Early ring	−24.3	1.2215 ± 0.0026	221.5 ± 2.6	39.0 ± 2.4	182.5 ± 5.0
68776	2010	Late ring	−24.5	1.0986 ± 0.0020	98.6 ± 2.0	40.0 ± 2.7	58.6 ± 4.7
68775	2010	Early ring	−25.0	1.1750 ± 0.0031	175.0 ± 3.1	40.0 ± 2.7	135.0 ± 5.8
4. Tomioka-1 (TOM-1, 37°20′33.0″N, 141°00′16.5″E, SSW 9 km of FDNPP)							
68782	2012	Whole ring	−24.4	1.0302 ± 0.0020	30.2 ± 2.0	31.5 ± 2.0	−1.3 ± 4.0
68783	2011	Late ring	−24.4	1.0594 ± 0.0022	59.4 ± 2.2	39.0 ± 2.4	20.4 ± 4.6
68784	2011	Early ring	−23.9	1.0378 ± 0.0022	37.8 ± 2.2	39.0 ± 2.4	−1.2 ± 4.6
68785	2010	Late ring	−24.0	1.0805 ± 0.0023	80.5 ± 2.3	40.0 ± 2.7	40.5 ± 5.0
68786	2010	Early ring	−24.3	1.1523 ± 0.0022	152.3 ± 2.2	40.0 ± 2.7	112.3 ± 4.9
5. Tomioka-2 (TOM-2, 37°20′33.0″N, 141°00′16.5″E, SSW 9 km of FDNPP)							
68792	2012	Whole ring	−24.5	1.0649 ± 0.0021	64.9 ± 2.1	31.5 ± 2.0	33.4 ± 4.1
68793	2011	Late ring	−24.4	1.0744 ± 0.0022	74.4 ± 2.2	39.0 ± 2.4	35.4 ± 4.6
68794	2011	Early ring	−24.3	1.0725 ± 0.0022	72.5 ± 2.2	39.0 ± 2.4	33.5 ± 4.6
68795	2010	Late ring	−26.1	1.0819 ± 0.0021	81.9 ± 2.1	40.0 ± 2.7	41.9 ± 4.8
68796	2010	Early ring	−24.7	1.0963 ± 0.0021	96.3 ± 2.1	40.0 ± 2.7	56.3 ± 4.8

^a^Data from [16]

**Fig. 2:**
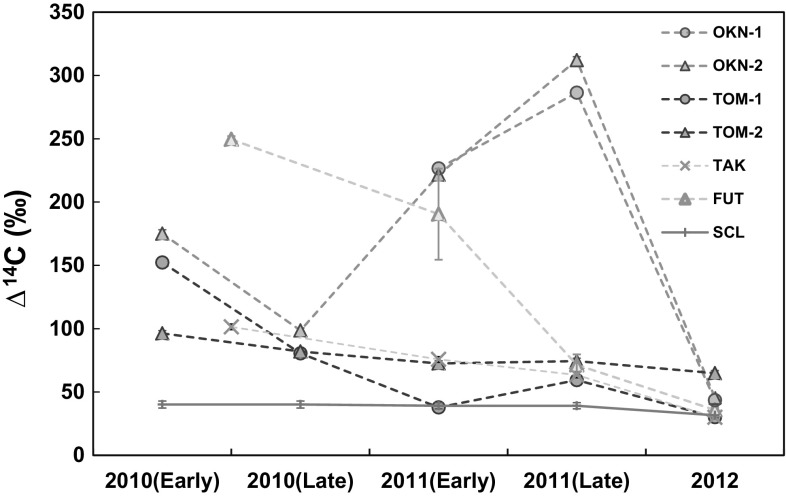
Sub-annual variations of Δ^14^C in tree-ring compared with the background value. The data of TAK and FUT was from [14], and the sub-unit data in the early wood and late wood from FUT was averaged to obtain the value for the whole early wood and late wood. The data from SCL was chosen as background [21]. The 1 σ uncertainties were within the marks

The sub-annual change in ^14^C activities of the tree rings in the vicinity of FDNPP from 2010 to 2012 are presented in Fig. 2. The data are from OKN and TOM (this study), as well as from Futuba (FUT), 2.5 km northwest of FDNPP), Takase (TAK), 11 km northwest of FDNPP) as given in [14], with SCL data for comparison. The ^14^C concentrations in OKN-1, 2 and TOM-2 2011 rings are obviously higher than the background in 2011. For the TOM-1 sample, there was excessive ^14^C in the 2011 late wood, although the ^14^C in early wood was the same with the background.

As described in [14], there are three possible causes of the ^14^C excess in the atmosphere: accumulation of the ^14^C discharged during normal operation of the reactors before the accident; the stored photosynthate from the previous year and the release from the accident. The first explanation has been excluded based on the relationship between excess ^14^C and electrical energy output [14]. The authors proved that the theoretical ^14^C discharge in 2011 before the accident was far lower than the observed value. Meanwhile, the issue of inheritance from the previous year has been argued, based on the rapid response of tree rings to the change in ^14^C concentration in the atmosphere. The contribution of stored photosynthate from the previous autumn is about 0–15% stem cellulose, which is relative low [11]. But considering the high ^14^C activity in the 2010 tree ring, this factor still cannot be negligible. In this study, the ∆^14^C in OKN samples had an evident increase from the late wood in 2010 to the early wood in 2011, reaching maximum values in the late wood of 2011. The nuclear plants were shut down after the accident and no additional ^14^C, caused by the normal operation of the plants entered the atmosphere after the shut-down. The continuous increase of ^14^C in OKN samples from the late wood in 2010 to the late wood in 2011 (Fig. 2) can only be explained by the release from the accident. This demonstrates that the released ^14^C indeed diffused southwards.

The two samples from OKN had higher ∆^14^C values in 2011 compared with previous data. The highest ∆^14^C value (312.1 ± 2.8‰) occurred in the OKN-2 sample. However, as we only have the data from two south-western locations, the total amount of ^14^C released and the diffusion range in the southwest cannot be quantified. It should be noted that even though the ∆^14^C value in OKN is higher, it still cannot compete with the release from the Chernobyl nuclear power plant (CNPP) accident which happened in 26th April 1986. The maximum excess ^14^C activity from FDNPP is 61.7 Bq kg^−1^ C (^14^C_excess_ = ∆^14^C_excess_/1000 × A_abs_, where the absolute radiocarbon standard (A_abs_) is defined as 226 Bq kg^−1^ C [22]), only about one fifth of the value from the CNPP accident (281.6 Bq kg^−1^ C) [23]. Such a difference could be accounted for the different operation principles of the two plants. The reactors in FDNPP are boiling-water reactor (BWR), where only trace amounts of ^14^N, ^13^C and ^17^O exist as impurities in the materials within the reactors [1, 16]. In contrast, ^13^C-rich graphite was used as the moderator in CNPP, which resulted in the ^13^C(n,γ)^14^C reaction within the reactor and subsequently a much higher ^14^C release after the accident [1, 23].

### The response of tree samples to the various atmospheric ^14^C concentration

In Fig. 2, the ∆^14^C variation between early wood and late wood in the 2011 tree ring samples from the southwest of FDNPP show a different trend to the ones from the northwest. In contrast to the sharp decrease in ∆^14^C from early wood to late wood of the 2011 tree rings at FUT (190.4 ± 36.0 to 71.4 ± 8.3‰) and TAK (75.7 ± 2.3 to 63.4 ± 2.3‰), the counterpart in OKN had an evident increase (226.6 ± 2.5 to 286.40 ± 2.6‰ for OKN-1 and 221.5 ± 2.6 to 312.1 ± 2.8‰ for OKN-2). This trend also existed in the TOM-1 sample (37.8 ± 2.2 to 59.4 ± 2.2‰), but not evident in the TOM-2 sample (72.5 ± 2.2 to 74.4 ± 2.2‰). The release of radionuclides from the accident was wide but temporary, and sharply decreased from late April [6]. Although there might be continuous low-volume release later, considering the main prevailing wind direction (mainly southeast from April to September [16]), it could not be the reason of the ∆^14^C increase in late wood. A similar study was taken previously to trace the release caused by the CNPP accident [13]. In their study, the early wood and late wood of 1986 tree ring samples were separated and analysed. The early wood and late wood in two samples were further split into two aliquots. The abnormally high ^14^C value was always present in the early wood and often in the first part of early wood, different from our results. The ∆^14^C peak in 2011 for the FUT sample existed a delay and was shown in the third part of the early wood [14]. Grooteset al. [11] reported a 5–6 week delay in ^14^CO_2_ incorporation into stem cellulose. This may account for the sub-annual change in the 2011 tree ring sample from FUT [14], but could not be used to the samples in this study. Kagawaet al. [24] reported a ^13^CO_2_ pulse-labelling test and proved a fast consumption of CO_2_ and incorporation of photosynthate within one month for *Cryptomeria japonica* in Japan. The transition between early and late wood is typically from mid-June and mid-July [25]. The accident would be too early to be recorded by the late wood. Alternatively, although no further radionuclide emissions were reported after this accident, the possibility that some ^14^CO_2_ was released after the accident (e.g., decommissioning process) when the wind direction was mainly southwest, or possible re-emission of radiocarbon from the surface soil near tree samples might be compatible with the regional distribution of the reversed trend. However, this hypothesis is difficult to prove because there is no available monitoring data showing possible release of other radionuclides during the decommissioning phase.

There is a slight variance between the two samples from TOM. The ^14^C activity in TOM-2 tree ring samples is relatively higher than that in TOM-1 from 2010 to 2012, except the early wood of the 2010 tree ring (Fig. 2). The TOM-2 sample recorded the evident ^14^C excess in 2011 for both early wood and late wood. For the TOM-1 sample, the ∆^14^C in the 2011 early wood is consistent with the annual background value, which is in contrast with the one in the late wood. The difference between these two samples might reflect the various plant physiologies of individual trees for response to the ^14^C in the atmosphere.

### The change of ^14^C activity in the atmosphere from 2010 to 2012

**Fig. 3:**
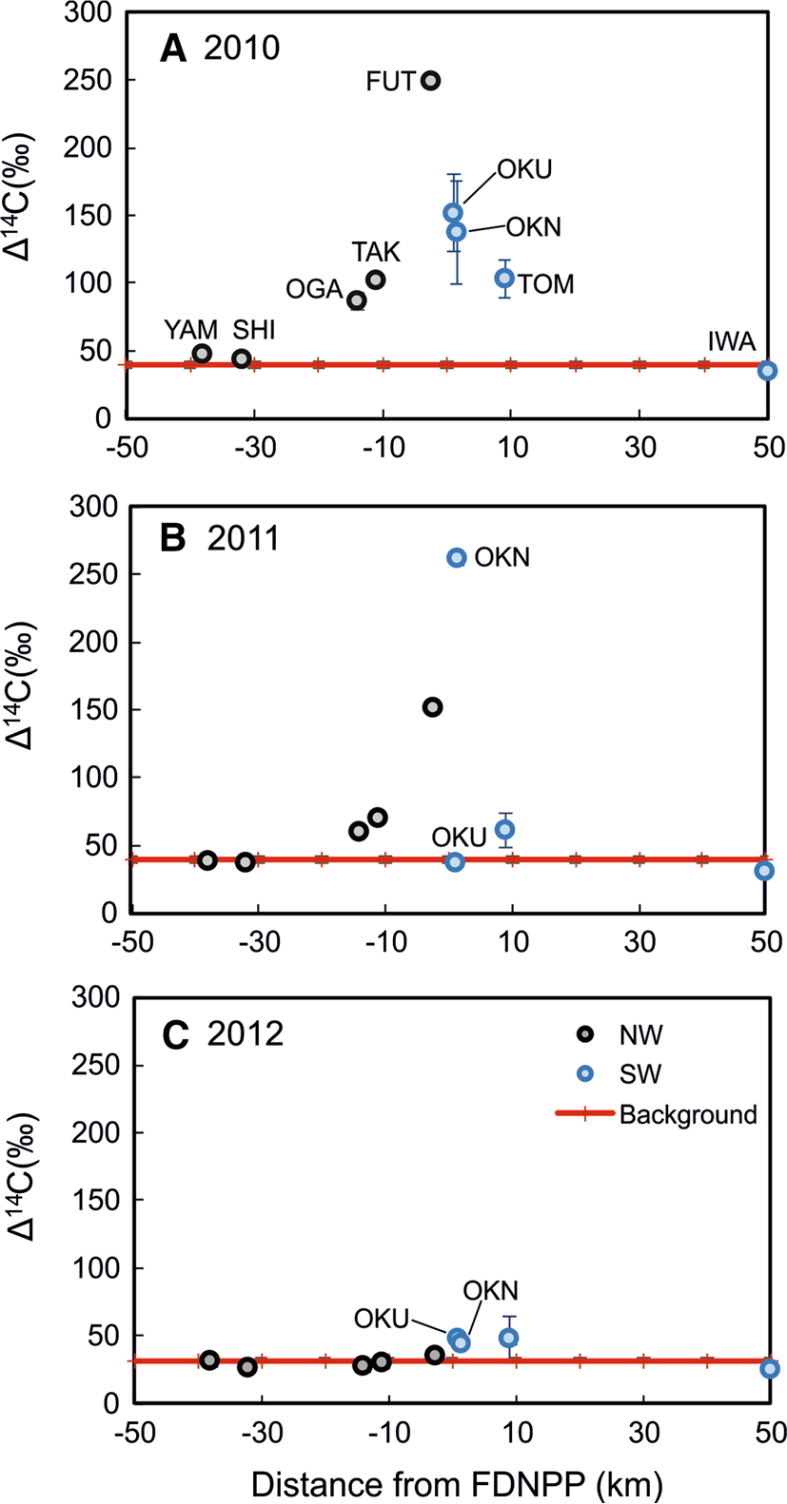
Annual change of Δ^14^C of tree ring samples in the vicinity of FDNPP from 2010 to 2012. The x-axis represnts the distance from FDNPP, the negative for NW and the positive for SW. The ∆^14^C data of early wood and late wood units from the same year was averaged to present the whole-year value. Then the annual data of the samples from the same location was averaged to obtain the whole year value for individual sampling location. The data of OKU was from [16]. The data of YAM, SHI, OGA, TAK and FUT was from [14]. The data from SCL was chosen as background [21]. The 1 σ uncertainties were shown in the figure

To examine the change in ^14^C caused by the accident, the annual amounts of ^14^C activities in tree ring between 2010 and 2012 are shown in Fig. 3. The ∆^14^C data of early wood and late wood units from the same year were averaged to present the whole-year value. Then, the annual data for the samples from the same location were averaged to obtain the whole year value for individual sampling location. In Fig. 3, the data from FUT, TAK, Ogaki (OKA, 14 km NW of FDNPP), Shimotsushima (SHI, 32 km NW of FDNPP), Yamakiya (YAM, 38 km NW of FDNPP) [14] were included for comparison.

In 2010, when the nuclear plant was in normal operation, there was a ∆^14^C excess in the atmosphere around FDNPP (Fig. 3a) and the influenced range reached 30 km in the northwest direction [14]. Research in the southwest is not comprehensive but the affected range is at least 8 km, based on this study. The peak activity was present in FUT rather than the closer site (OKU), which could be due to the dominant southeast wind over the whole year.

The exposed area of the accident is reduced to 14 km wide in the northwest, but the same in the southwest (Fig. 3B). For the short release time, ∆^14^C in 2011 was relatively lower than that in 2010, except in OKN samples. Although they recorded the highest ^14^C peak, as the distance of OKN from FDNPP is closer than that of FUT, we cannot preclude higher values existing in the northwest. The result of duplicate OKU sample analyses revealed no excess observed in both early wood and late wood units in 2011. The data was averaged to present the annual value (37.4 ± 2.0‰) which is in good agreement with the published result (40.3 ± 2.0‰) [16] within the error range. This demonstrates good reproducibility in our studies, and the tree sample from OKU indeed failed to record the accident. But the ^14^C excess was clearly visible in the OKN trees which are in the same direction and only 1 km from OKU. This abnormal phenomenon might be due to the plant physiology, but no conclusive explanation can be made.

After the accident, the FDNPP was abandoned, resulting in the sharp decrease in ^14^C activity in the atmosphere (Fig. 3c). While north-western samples were similar to the background, there was a slight ^14^C excess in southern samples. At present, we have no convincing explanations for this ^14^C excess. We presume that it might be caused by the localised high ^14^C value in the atmosphere, as demonstrated by the inter-annual variation in ^14^CO_2_ observed at Ohkuwa [26]. Alternatively, it could also be attributed to a potential release of ^14^C during the decommissioning of FDNPP. However, the limited data constrained us from reaching an exclusive conclusion. More sampling work will be conducted to clarify these alternatives in the future.

## Conclusions

In this study, we determined the sub-annual and annual variability in the ^14^C value of the atmosphere near the FDNPP from 2010 to 2012. The discovery of ^14^C excess in new 2011 tree ring samples from OKN and TOM demonstrated southwest dispersion of the ^14^C released during the accident. The measured maximum amount of ^14^C was present in the late wood of 2011 from sample OKN-2. The reversed increase of ^14^C concentration from the early wood to the late wood in 2011 in this study implied possible release of ^14^C nearby after the accident. However, more research is needed to testify this assumption. Furthermore, more studies are required to explain the failure of tree ring sample from Okuma to record the release of ^14^C and the abnormal excess of ^14^C in the 2012 tree ring samples from southwest of FDNPP.
